# Report of Poisoned Wounds

**Published:** 1855-01

**Authors:** 


### Reviews and Bibliographical Notices.

**REPORT OF POISONED WOUNDS. By A. F. Geeter, M. D., Palmyra, Mo.**— To the Medical Association of Missouri.

This is a very interesting pamphlet, and well written. The Dr. has experimented largely with the poison of serpents, and his views well sustained by observation and facts. May not the rapid and great swelling depend upon the transudation of the watery parts of the blood into the cellular tissue, while the capillary circulation is in a state of stasis, and the color the result also of a congestion of those vessels? The blood in the subcutaneous vessels, found to be coagulated, indicates this loss; the fibrin and the more solid constituents remaining within them.

The pamphlet is a valuable contribution, and exhibits a mind imbued with the importance of research, and an ability to prosecute it. Will the author continue his enquiries?

